# The Autoinducer N-Octanoyl-L-Homoserine Lactone (C8-HSL) as a Potential Adjuvant in Vaccine Formulations

**DOI:** 10.3390/ph16050713

**Published:** 2023-05-08

**Authors:** Sarthak M. Shah, Devyani Joshi, Christiane Chbib, Monzurul A. Roni, Mohammad N. Uddin

**Affiliations:** 1 Department of Pharmaceutical Sciences, College of Pharmacy, Mercer University, Atlanta, GA 30341, USA; 2 Department of Pharmaceutical Sciences, College of Pharmacy, Larkin University, Miami, FL 33169, USA; 3 Department of Health Sciences Education and Pathology, University of Illinois College of Medicine, Peoria, IL 61605, USA

**Keywords:** C8-HSL microparticle, autoinducers, adjuvant, vaccines, immunogenicity

## Abstract

Autoinducers AI-1 and AI-2 play an important role in bacterial quorum sensing (QS), a form of chemical communication between bacteria. The autoinducer N-octanoyl-L-Homoserinehomoserine lactone (C8-HSL) serves as a major inter- and intraspecies communicator or ‘signal’, mainly for Gram-negative bacteria. C8-HSL is proposed to have immunogenic properties. The aim of this project is to evaluate C8-HSL as a potential vaccine adjuvant. For this purpose, a microparticulate formulation was developed. The C8-HSL microparticles (MPs) were formulated by a water/oil/water (W/O/W) double-emulsion solvent evaporation method using PLGA (poly (lactic-co-glycolic acid)) polymer. We tested C8-HSL MPs with two spray-dried bovine serum albumin (BSA)-encapsulated bacterial antigens: colonization factor antigen I (CFA/I) from *Escherichia coli* (*E. coli*.) and the inactive protective antigen (PA) from *Bacillus anthracis* (*B. anthracis*). We formulated and tested C8-HSL MP to determine its immunogenicity potential and its ability to serve as an adjuvant with particulate vaccine formulations. An in vitro immunogenicity assessment was performed using Griess’s assay, which indirectly measures the nitric oxide radical (NOˑ) released by dendritic cells (DCs). The C8-HSL MP adjuvant was compared with FDA-approved adjuvants to determine its immunogenicity potential. C8-HSL MP was combined with particulate vaccines for measles, Zika and the marketed influenza vaccine. The cytotoxicity study showed that MPs were non-cytotoxic toward DCs. Griess’s assay showed a comparable release of NOˑ from DCs when exposed to CFA and PA bacterial antigens. Nitric oxide radical (NOˑ) release was significantly higher when C8-HSL MPs were combined with particulate vaccines for measles and Zika. C8-HSL MPs showed immunostimulatory potential when combined with the influenza vaccine. The results showed that C8-HSL MPs were as immunogenic as FDA-approved adjuvants such as alum, MF59, and CpG. This proof-of-concept study showed that C8-HSL MP displayed adjuvant potential when combined with several particulate vaccines, indicating that C8-HSL MPs can increase the immunogenicity of both bacterial and viral vaccines.

## 1. Introduction

Adjuvants are compounds that enhance the stability and immunogenicity of vaccine antigens, modulate the efficacy, and increase the level of immune responses to the antigen [1]. There are several proposed mechanisms by which adjuvants act to enhance the immunogenicity of vaccines: (1) adjuvants can create a depot effect, which allows slow release of antigens from the immunization site [2]; (2) adjuvants stimulate the secretion of cytokines and chemokines; (3) adjuvants cause cellular infiltration at the site of injection; and (4) adjuvants enhance antigen uptake and presentation to APCs. In this process, adjuvants directly or indirectly act as an antigen delivery system that increases phagocytosis and pinocytosis, leading to enhanced APC antigen uptake. For example, adjuvant MF59 increases the number of APCs in draining lymph nodes in comparison to alum [3]. Additionally: (5) adjuvants initiate the activation and maturation of APCs by increasing MHC class II and costimulatory molecule expression; and (6) adjuvants activate inflammasomes [1]. For example, alum adjuvant activates the NLRP3 inflammasome [4]. Adjuvants increase the speed and duration of the immune response, stimulate cytotoxic T lymphocytes, and improve the immune response in immune-compromised adults, newborns, and elderly patients. In this way, adjuvants reduce the dose of vaccine antigens and number of administrations needed for protective immunity, while increasing antibody avidity and specificity [5]. Both synthetic and biomolecules can be used as vaccine adjuvants. Based on the mechanism of their effect, adjuvants are generally categorized into two groups: immunostimulatory adjuvants and vaccine delivery systems. Immunostimulatory adjuvants such as immunostimulatory complexes that represent a supramolecular combination of saponins from *Quillaja saponaria*, cholesterol, and phosphatidylcholine [6], mineral salts, microorganism-derived compounds, etc., directly contribute to enhancing the immune response. However, vaccine delivery systems, such as liposomes and micro-nanoparticles, contribute to the enhanced response due to their unique delivery properties [5].

**Figure 1:**
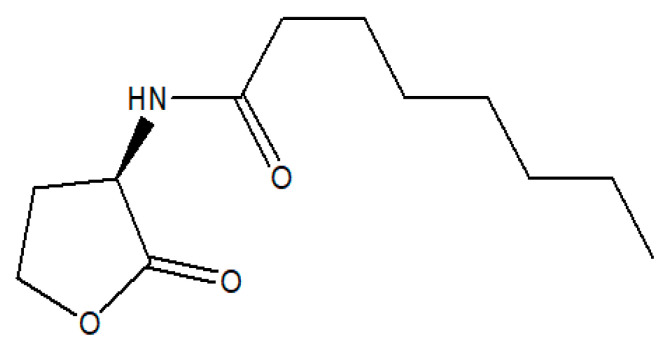
Chemical structure of C8-HSL, N-octanoyl-L-homoserine lactone.

In this research project, we evaluated the bacterial biomolecule N-octanol-L-homoserine lactone, or autoinducer-1 (C8-HSL), as a potential vaccine adjuvant in vaccine formulations. Bacteria can communicate via different signals or inducers using autoinducer biomolecules that serve as a common communicator or ‘signal’ of both Gram-positive and Gram-negative bacteria. Autoinducer signals mediate inter- and intra-species quorum sensing (QS) systems among different bacteria by coordinating gene expression of population above a certain cell density [7]. In addition to controlling the bacterial population, these signals also can activate the APC by appearing as a foreign pathogen. Once these signals reach a threshold, it leads to a cumulative dendritic cell activation [8]. Autoinducer-1 (C8-HSL) (Figure 1) serves as a common communicator among Gram-negative bacteria [9]. This molecule is a member of the N-acyl-homoserine lactone family. Previously, DPD, a precursor of autoinducer-2, was determined to be immunogenic and was shown to have adjuvant potential [5]. To further investigate, in this research project, we have continued the study with the biomolecule C8-HSL as a vaccine adjuvant. Since the C8-HSL compound is a common “signal” for bacterial communication, we hypothesized that this compound can be used as an adjuvant in vaccine formulations. One of the efficient formulations is a particulate vaccine formulation. Particulate formulation has numerous benefits; particles can serve as antigen carriers, provide controlled release of antigen, modulate immune responses, and protect the integrity of antigens from degradation in the GI tract [10].

For example, microparticles loaded with ovalbumin (OVA) have been shown to have immunogenicity equivalent to that of OVA with Freund’s adjuvant (water-in-oil emulsion of heat-killed M. tuberculosis) [11]. This confirms the use of particulate vaccine delivery systems as adjuvants [12,13,14,15]. In this study, we have used particulate vaccine formulation of C8-HSL. Formulating C8-HSL into a microparticle can offer several advantages. It may express adjuvant effects, and offer controlled antigen release, leading to mounting a stronger immune response [11,16,17,18].

In addition to developing the particulate vaccine formulation, we also evaluated the cytotoxicity of C8-HSL MP toward antigen-presenting cells (APCs) using Griess’s assay [19,20]. We also investigated the immunological profile of this C8-HSL microparticulate compound and its potential for serving as an adjuvant in particulate vaccine formulations. The immunostimulatory potential of microparticulate vaccines was compared with that of current marketed FDA-approved microparticulate formulations such as Alhydrogel^®^ (alum), AddaVax™ (MF59), and cytosine-phosphorothioate-guanine oligodeoxynucleotides (CpG) [21]. We further tested our compound for adjuvant potential against viral antigens such as inactivated Zika vaccine MP, measles vaccine MP, and the marketed influenza vaccine.

## 2. Results

### 2.1. Characterization of Antigen and Adjuvant Microparticles

**Figure 2:**
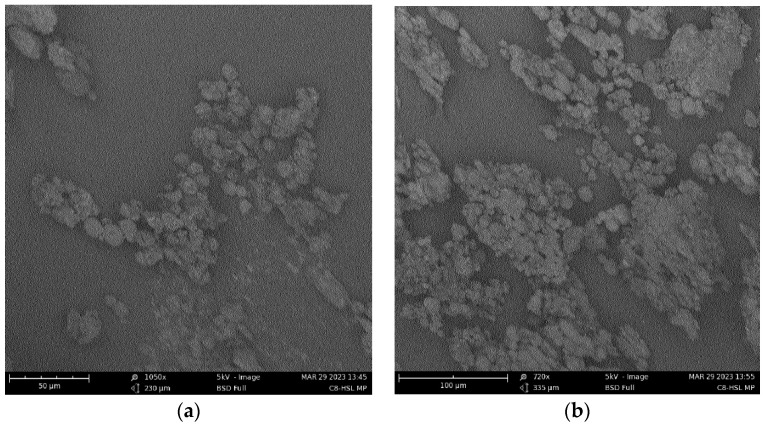

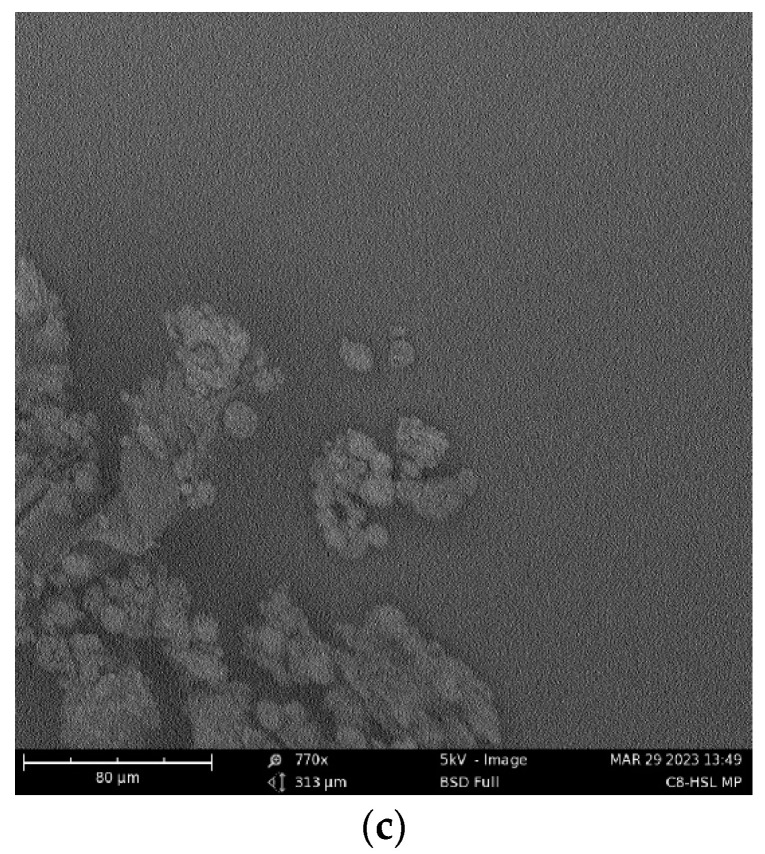
Scanning electron microscopy (SEM) of autoinducer C8-HSL microparticles. The C8-HSL MPs were spherical with a smooth surface. (**a**–**c**) are showing the images of different areas of the same sample.

MPs were characterized by particle size, charge, and polydispersity index. The surface morphology was visualized by scanning electron microscopy. The recovery yield of our experimental adjuvant, C8-HSL, was a high product yield (74%). For all adjuvants, a high recovery yield (70–90%) was obtained. For the antigens, a high recovery yield of 74–86% was obtained. Using a laser particle counter, it was determined that there were 1032 ± 10 particles of C8-HSL/mL (1 mg/mL). The particle size ranged from 2–5 μm. Generally, microparticles that are in the range of 1 and 5 μm are phagocytosed better by antigen-presenting cells [22]. The polydispersity index (PDI) of our experimental adjuvant, C8-HSL MP, was 0.468 ± 0.345. The PDI of all adjuvant MPs ranged from 0.4 to 0.6, while the PDI of all antigen MPs ranged from 0.3 to 0.5. The zeta potential or charge of C8-HSL MP was −32.0 ± 0.92 mV. The charge of all adjuvant MPs ranged from −18 to −32 mV. The charge of all antigen MPs ranged from −19 to −32 mV. The recovery yield, particle size, polydispersity index (PDI), and zeta potential of the adjuvant and antigen microparticles are indicated in Table 1. The low PDI indicated a uniform size distribution of the particles. The negative surface charge indicated the high stability of MP in an aqueous solution. The morphology (Figure 2) of the particles was observed using a Phenom™ benchtop scanning electron microscope (SEM). The particles were found to be spherical in SEM images.

### 2.2. Autoinducer C8-HSL Microparticulate Concentration Optimization

**Figure 3:**
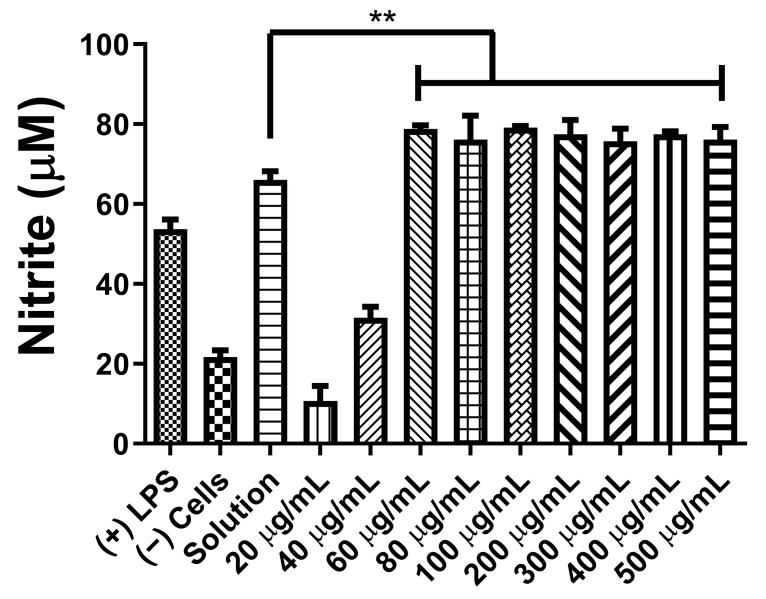
Griess assay for NOˑ release from dendritic cells upon exposure to different concentrations of the MPs. Lipopolysaccharide (50 µg/mL) and cells only served as positive and negative controls, respectively. DC cells were exposed to several concentrations of C8-HSL MPs ranging from 20 μg/mL to 500 μg/mL. The incubation time was 24 h. C8-HSL MP expressed a high NOˑ release (*p* < 0.01) at 60 μg/mL. This test was replicated in triplicate. *p* ≤ 0.01 (**). In all experiments, a *p*-value of less than 0.05 was considered statistically significant.

The NOˑ released from C8-HSL MP-stimulated dendritic cells was measured by the Griess assay. Different concentrations were used to determine the NOˑ release upon microparticle exposure. The data are shown in Figure 3.

### 2.3. Cytotoxicity Profile of C8-HSL MPs

**Figure 4:**
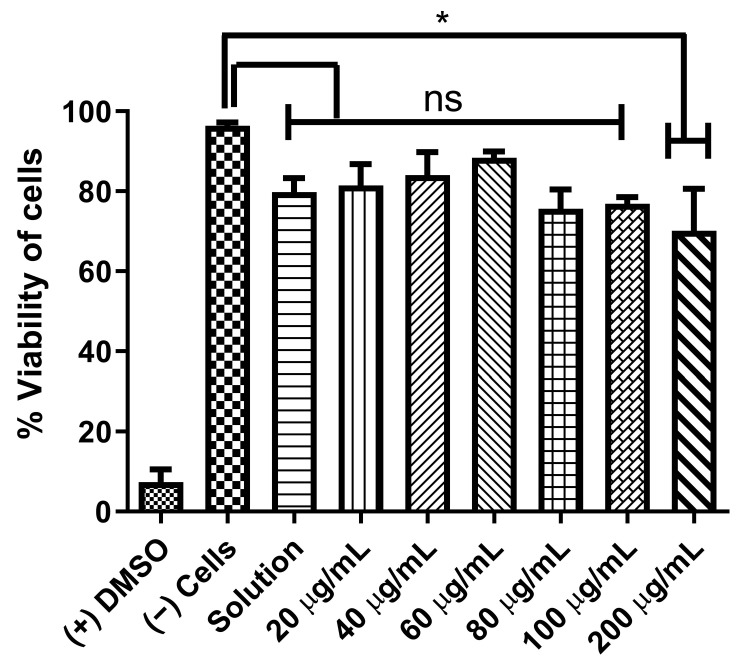
Cytotoxicity study of C8-HSL MP. The percentage viability of cells of C8-HSL MP was assessed via the MTT assay. The cell density of Dendritic 2.4 cells (DCs) was 3.5 × 10^5^ cells/well. The cells were pulsed with increasing concentrations of C8-HSL MPs (20 μg/mL to 500 μg/mL) at a volume of 100 μL/well or with DMSO (50 μL/well). The cells were incubated at 37 °C for 24 h. DMSO and the cells only group served as controls in this experiment. C8-HSL MPs were not cytotoxic to dendritic cells (*p* ≤ 0.05). This test was replicated in triplicate. *p* > 0.05 (ns, non-significant), *p* ≤ 0.05 (*). In all experiments, a *p*-value of less than 0.05 was considered statistically significant.

The cytotoxicity of the autoinducer C8-HSL MP was determined using dendritic cells. This experiment was conducted in vitro utilizing the MTT assay. The toxic solvent dimethyl sulfoxide (DMSO) was used as a positive control to kill the cells. On the other hand, cell lines without the addition of C8-HSL were used as negative controls. The dendric cells were challenged with different concentrations of C8-HSL to evaluate cytotoxicity. C8-HSL was found to be non-cytotoxic towards DCs from low concentration (20 μg/mL) to high concentration (200 μg/mL). The data are shown in Figure 4.

### 2.4. In Vitro Release Study of C8-HSL

**Figure 5:**
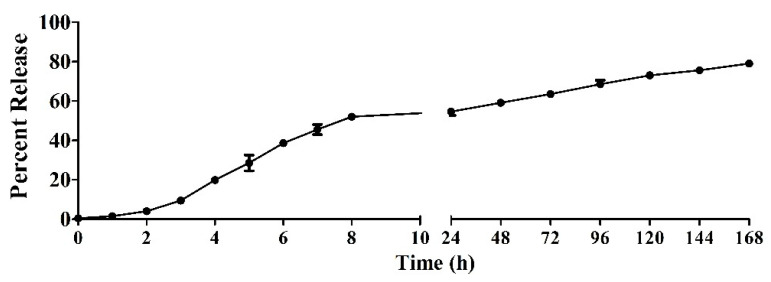
In vitro release study of C8-HSL from PLGA polymer in phosphate buffer saline (PBS) at pH 7.4, 37 °C. The release study was conducted for 168 h (7 d). Data are represented as the mean ± SEM. A 50% release of C8-HSL was observed from the PLGA polymer at 7 h.

A release study of C8-HSL was conducted to determine the amount of C8-HSL that is released from the PLGA polymer for up to 168 h (7 days). In the first 7 h, approximately 50% of C8-HSL was released. Afterwards, the release of C8-HSL was very slow. The release study data are shown in Figure 5.

### 2.5. Evaluation of C8-HSL MP Induction of Autophagosomes

**Figure 6:**
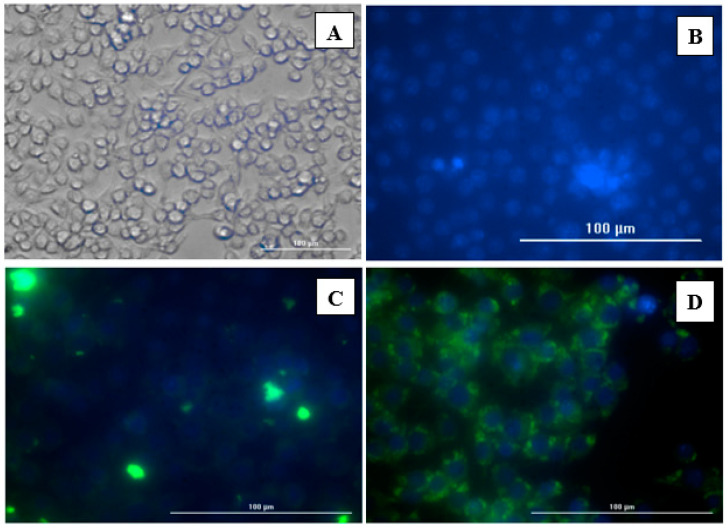
Induction of autophagosomes was observed using the autophagy assay. The green fluorescence protein-conjugated light chain 3 (GFP-LC3 puncta) is representative of active autophagy, while the blue (DAPI, 1 mg/mL) represents the nucleus staining. (**A**) dendritic cells, (**B**) rapamycin inhibitor, (**C**) PA antigen (60 μg/mL), and (**D**) PA antigen (30 μg/mL) + C8-HSL MPs (30 μg/mL).

Autophagy has been shown to facilitate antigen processing to major histocompatibility complexes (MHCs) I and II to alert CD4^+^ and CD8^+^ T cells to an infection [23,24]. Autophagy is important for antigen cell presentation via major histocompatibility complex I or II. There were four groups in the autophagy study: no treatment group (dendritic cells only), inhibitor group (rapamycin), antigen group (PA MP antigen), and antigen plus adjuvant group (PA MP antigen + C8-HSL MP). Green dye is specifically for autophagosomes. This green dye is a fluorescence protein-conjugated light chain 3 (GFP-LC3 puncta) that represents active autophagy. The PA MP combined with the C8-HSL MP group showed significantly higher expression of autophagosomes than the PA antigen MPs. The results of autophagy in a live cell imager Biotek are shown in Figure 6.

### 2.6. Immunostimulatory Potential of C8-HSL Microparticles

**Figure 7:**
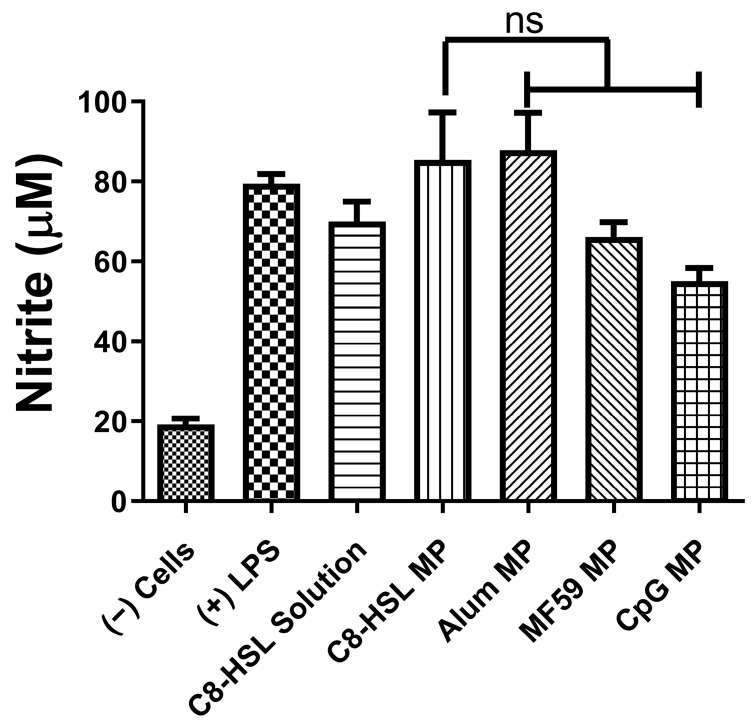
Immunostimulatory potential of microparticles: Experimental adjuvants C8-HSL MP, Alum MP, MF59 MP, and CpG MP were exposed to dendritic cells at 60 μg/mL. Lipopolysaccharide (50 µg/mL)-stimulated and unstimulated dendritic cells served as positive and negative controls, respectively. Data are represented as the mean ± SEM. No significant difference (*p* > 0.05) was found between C8-HSL MP and other adjuvants. This test was replicated in triplicate.

**Figure 8:**
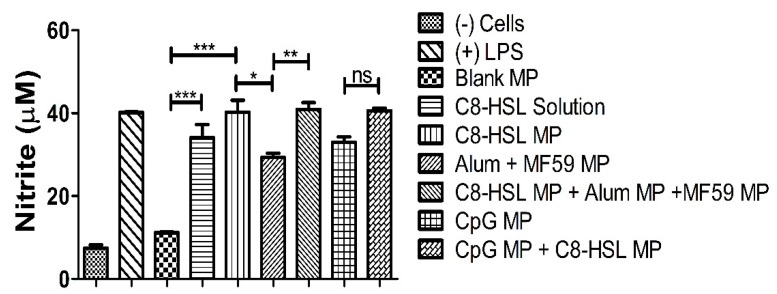
Adjuvant combination study. Lipopolysaccharide (LPS, 50 μg/mL) and cells only served as positive and negative controls, respectively. DCs were harvested in a 48-well plate and exposed to vaccine particles overnight for 24 h. Blank MP, C8-HSL solution, and C8-HSL MP had a dose of 60 μg/mL. The group consisting of 3 adjuvants had a dose of 20 μg/mL each. The group consisting of CpG MP and C8-HSL MP had a dose of 30 μg/mL each. One-way ANOVA and Tukey’s post hoc tests were performed. Note: *p* > 0.05 (ns, nonsignificant), *p* ≤ 0.05 (*), *p* ≤ 0.01 (**), *p* ≤ 0.001 (***). In all experiments, a *p* value of less than 0.05 was considered statistically significant. This test was replicated in triplicate.

The immunostimulatory potential of C8-HSL MP was compared with that of the FDA-approved adjuvants alum, MF59, and CpG. Nitrite oxide release from dendritic cells after stimulation for 24 h was quantified using the Griess assay [5]. In addition, NOˑ release was compared when C8-HSL was combined with other adjuvants, to observe the combination effect. In this study, DCs were harvested in a 48-well plate and exposed to vaccine particles (dose = 60 µg/µL). C8-HSL MP was found to be immunostimulatory, similar to FDA-approved adjuvants (*p* < 0.001). The release of NOˑ was significantly higher when C8-HSL was combined with alum, MF59, and CpG MPs. Based on our results (Figure 7), C8-HSL MPs were significantly more immunostimulatory than blank MP (*p* ≤ 0.001). C8-HSL MP + Alum MP + MF59 MP were more immunogenic (*p* ≤ 0.01) than C8-HSL MP. However, there was no significant difference observed when C8-HSL MP was combined with CpG adjuvant. These results indicate that C8-HSL MP can be combined with Alum MP and MF59 MP (Figure 8). Recent literature suggests that MF59 is a more potent immunostimulatory adjuvant than CpG. Additionally, MF59 was found to induce the release of extracellular ATP from muscle cells, triggering endogenous stress signals that can aid in activating the innate immune system [25]. One-way ANOVA and Tukey’s post hoc tests were performed for statistical analysis.

### 2.7. Evaluation of Adjuvant Effect with Bacterial Antigens: CFA and PA Antigens

**Figure 9:**
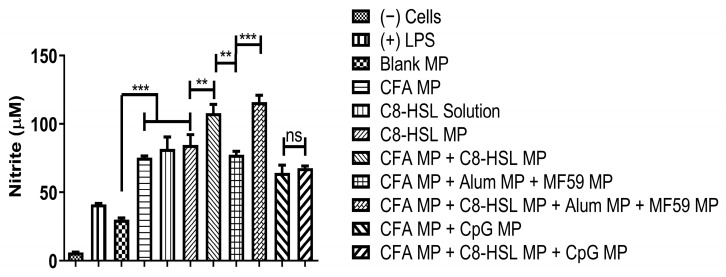
Evaluation of the adjuvant effect of C8-HSL MP and FDA-approved adjuvants with CFA antigen. DCs were seeded in a 48-well plate and exposed to vaccine particles overnight for 24 h. Lipopolysaccharide (LPS, 50 μg/mL) and cells only served as positive and negative controls, respectively. C8-HSL solution and C8-HSL MP had a dose of 60 μg/mL. The group consisting of CFA MP and 2 adjuvants had a dose of 20 μg/mL each. The group consisting of CFA MP and 3 adjuvants had a dose of 15 μg/mL each. There was a significant difference when C8-HSL MP was combined with CFA MP (*p* ≤ 0.01). There was a significant difference when C8-HSL MP was combined with CFA MP + Alum MP + MF59 MP (*p* ≤ 0.001). There was no significant difference when C8-HSL MP was combined with CFA MP + CpG MP (*p* > 0.05). This test was replicated in triplicate. Note: *p* > 0.05 (ns, nonsignificant), *p* ≤ 0.01 (**), *p* ≤ 0.001 (***). In all experiments, a *p* value of less than 0.05 was considered statistically significant.

**Figure 10:**
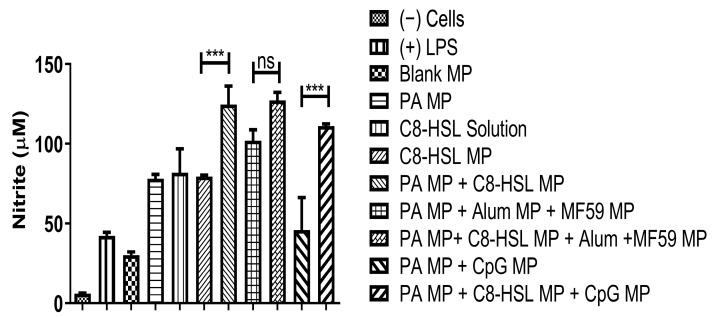
Evaluation of the adjuvant effect of C8-HSL MP and FDA-approved adjuvants with PA antigen. DCs were harvested in a 48-well plate and exposed to vaccine particles overnight for 24 h. Lipopolysaccharide (LPS, 50 μg/mL) and cells only served as positive and negative controls, respectively. C8-HSL solution and C8-HSL MP had a dose of 60 μg/mL. The group consisting of PA MP and 2 adjuvants had a dose of 20 μg/mL each. The group consisting of PA MP and 3 adjuvants had a dose of 15 μg/mL each. C8-HSL MP showed an adjuvant effect when combined with the bacterial antigen PA (Protective Antigen) (*p* < 0.001). There was a significant difference when C8-HSL MP was combined with PA MP (*p* ≤ 0.001). There was a significant difference when C8-HSL MP was combined with PA MP + CpG MP (*p* ≤ 0.001). There was no significant difference when C8-HSL MP was combined with PA MP + Alum MP + MF59 MP (*p* > 0.05). This test was replicated in triplicate. Note: *p* > 0.05 (ns, nonsignificant), *p* ≤ 0.001 (***). In all experiments, a *p* value of less than 0.05 was considered statistically significant.

The release of nitric oxide by dendritic cells was measured when C8-HSL MP was combined with two antigens. The first antigen is colonization factor antigen I MP (CFA/I) from *E. coli*, and the second is protective antigen (PA) MP from inactivated *Bacillus anthracis*. C8-HSL MP, when combined with CFA MP and PA MP antigen, displayed significantly higher NOˑ release when compared to the blank MPs. LPS and cells only served as positive and negative controls, respectively. DCs were harvested in a 48-well plate and exposed to vaccine particles (dose = 60 µg/µL). C8-HSL MP showed an adjuvant effect when combined with the bacterial antigen CFA colonization factor antigen I (*p* < 0.001). One-way ANOVA and Tukey’s post hoc tests were performed for statistical analysis. The results of the adjuvant effect of C8-HSL MP and FDA-approved adjuvants with CFA antigen are shown in Figure 9. The results of the adjuvant effect of C8-HSL MP and FDA-approved adjuvants with PA antigen are shown in Figure 10.

### 2.8. Evaluation of Adjuvant Effect with Viral Antigens: Zika, Measles, and the Marketed Influenza Vaccine

**Figure 11:**
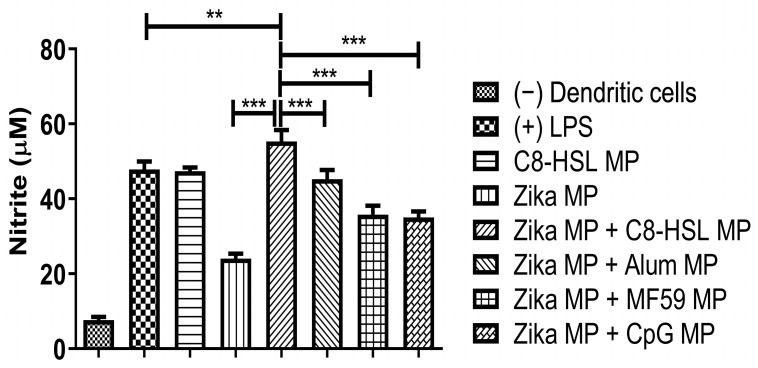
Evaluation of the adjuvant effect of C8-HSL MP and FDA-approved adjuvants (Alum MP, MF59 MP, and CpG MP) with the Zika vaccine MP. Concentrations: Lipopolysaccharide (LPS, 50 μg/mL) and cells only served as positive and negative controls, respectively. C8-HSL MP and Zika MP had a dose of 60 μg/mL. The Zika MP + one adjuvant group had a dose of 30 μg/mL each. There was a significant difference when C8-HSL MP was combined with Zika MP (*p* ≤ 0.001). Zika MP + C8-HSL MP showed a significant difference versus Zika MP + other adjuvants (*p* ≤ 0.001). This test was replicated in triplicate. Note: *p* ≤ 0.01 (**), *p* ≤ 0.001 (***). In all experiments, a *p* value of less than 0.05 was considered statistically significant.

**Figure 12:**
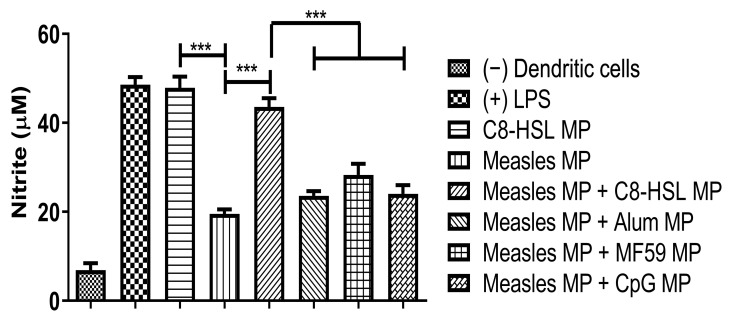
Evaluation of the adjuvant effect of C8-HSL MP and FDA-approved adjuvants (alum, MF59, and CpG) with the Zika vaccine MP. Concentrations: Lipopolysaccharide (LPS, 50 μg/mL) and cells only served as positive and negative controls, respectively. C8-HSL MP and measles MP had a dose of 60 μg/mL. The measles MP + one adjuvant group had a dose of 30 μg/mL each. There was a significant difference when C8-HSL MP was combined with measles MP (*p* ≤ 0.001). Measles MP + C8-HSL MP showed a significant difference versus measles MP + other adjuvants (*p* ≤ 0.001). This test was replicated in triplicate. Note: *p* ≤ 0.001 (***). In all experiments, a *p* value of less than 0.05 was considered statistically significant.

**Figure 13:**
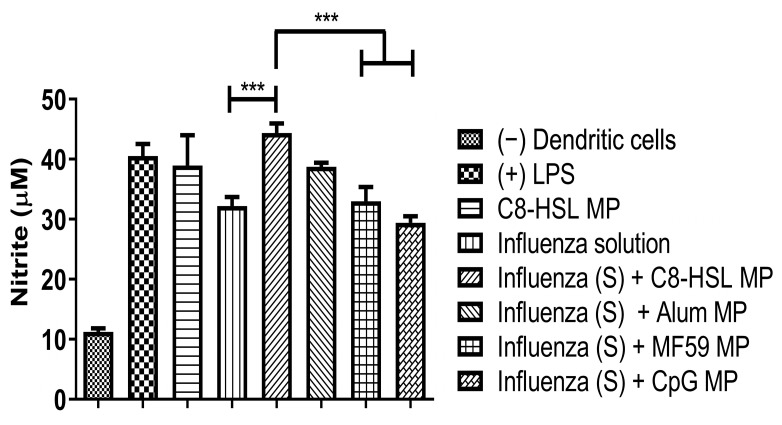
Evaluation of the adjuvant effect of C8-HSL MP and FDA-approved adjuvants (alum, MF59, and CpG) with the marketed influenza vaccine. Concentrations: Lipopolysaccharide (LPS, 50 μg/mL) and cells only served as positive and negative controls. C8-HSL MP and influenza solution had a dose of 60 μg/mL. The influenza solution + one adjuvant group had a dose of 30 μg/mL each. There was a significant difference when C8-HSL MP was combined with marketed influenza vaccine (*p* ≤ 0.001). There was a significant difference when the C8-HSL MP was combined with the influenza vaccine versus other adjuvants (*p* ≤ 0.001). Note: *p* ≤ 0.001 (***). In all experiments, a *p* value of less than 0.05 was considered statistically significant.

Release of nitric oxide by dendritic cells was measured when C8-HSL MP was combined with three vaccines: two microparticulate (Zika and measles), and one marketed (influenza). Figure 11 shows NOˑ release when C8-HSL MP is combined with the Zika MP vaccine versus when the Zika MP vaccine is combined with FDA-approved adjuvants (Alum MP, MF59 MP, and CpG MP). Figure 12 shows NOˑ release when C8-HSL MP is combined with the measles MP vaccine versus when the measles MP vaccine is combined with FDA-approved adjuvants (Alum MP, MF59 MP, and CpG MP). Figure 13 shows NOˑ release when C8-HSL MP is combined with a marketed influenza vaccine versus when this vaccine is combined with FDA-approved adjuvants (Alum MP, MF59 MP, and CpG MP).

## 3. Discussion

Bacterial communication takes place using chemical signaling molecules called autoinducers, which regulate bacterial gene expression in a process known as quorum sensing [5]. Quorum sensing (QS) is a process in which bacteria communicate using various signals or inducers [26]. The bacteria can mount a strong collective response through the exchange of small chemical signals. QS results in higher tolerance of these bacteria to antibiotics, which results in bacterial resistance [27]. Autoinducer molecules serve as a common communicator or “signal” of both Gram-positive and Gram-negative bacteria. Once this “signal” reaches a threshold, cumulative dendritic cell activation occurs [26]. Autoinducers can also activate the antigen presenting cells such as dendritic cells. Autoinducers activate the dendritic cell (DC) through the prolonged exposure to the TLR3 and MDA-5 receptors that lead to the initiation of antigen immunity.

Previously, we studied the potential adjuvant effect of a microparticulate formulation of the (S)-4,5-dihydroxy-2,3-pentanedione (DPD) molecule, a precursor of autoinducer-2. In this study, the adjuvant effect of (S)-4,5-dihydroxy-2,3-pentanedione (DPD) was tested by combining it with particulate vaccines for measles and gonorrhea and comparing the adjuvant effect observed with the microparticulate formulations of the FDA-approved adjuvants alum, MPL A^®^, and MF59^®^. The results showed that microparticulate (S)-DPD was noncytotoxic toward antigen-presenting cells and had an adjuvant effect with the microparticulate gonorrhea vaccine [5]. Since the C8-HSL compound is a common “signal” for bacterial communication, we hypothesized that it may also have an adjuvant effect. Therefore, we attempted to investigate its adjuvant potency. The microparticulate formulation of C8-HSL helps to increase its immunostimulatory effects [5,12,19]. We studied the immunological profile of this microparticulate compound and its potential for serving as an adjuvant in vaccine formulations. PLGA was chosen as a polymer for microparticulate formulation owing to its ability to induce controlled release, enhance humoral and cellular immune responses, and increase antigen uptake by APCs [28]. Numerous studies suggest that small PLGA particles can produce strong or potent immune responses [29]. Studies have shown that in certain sizes, PLGA are the most biologically active in antigens presenting cell immune response [30]. Therefore, we have characterized the PLGA microparticles in terms of their size, polydispersity index (PDI), and surface charge. PLGA microparticles generally show a low PDI, indicating a uniform size distribution of the particles [2,30]. The negative surface charge indicates the high stability of MP in an aqueous solution. Generally, a higher positive or negative charge (mV) is favored for colloidal suspensions to prevent clumping of particles [19]. In our study we have used carboxylic acid terminal PLGA. The negative charge of the particle originates from carboxylic acid. Previously it has been found that the terminal carboxyl groups on the surface decrease the zeta potentials. In general, surfactant-free PLGA NPs exhibit a negative zeta potential (−49 mV) due to the presence of terminal carboxylic groups in PLGA [31]. Morphologically, the C8-HSL microparticles were spherical. Spherical shape indicates better antigen uptake by antigen-presenting cells (APCs) [32,33]. Moreover, the release study confirmed a sustained release of C8-HSL from microparticles. Almost 50% release of C8-HSL from the polymer occurred at approximately 7 h (Figure 5). This release pattern of C8-HSL from PLGA is similar to reported values in the literature [19,34]. Additionally, the safety profile of microparticles is crucial to developing a safe vaccine. A cytotoxicity study indicated that C8-HSL MPs are noncytotoxic at increasing concentrations of up to 200 µg/mL when exposed to dendritic cells. Evaluation of in vitro immunostimulatory potential was conducted by measuring nitric oxide (NOˑ) released by DC 2.4 using the Griess assay. Once an antigen or foreign pathogen is recognized by antigen-presenting cells such as dendritic cells and macrophages, they release a non-specific innate immune marker NOˑ and other cytokines as IL-12, TNF, IL-6, and IFN-γ [35]. The release of NOˑ from APCs allows the recruitment of additional APCs and other immune cells. The additional reinforcement of other immune cells allows the immune system to produce a significant immune response to the foreign pathogen [36,37]. Quantifying the release of NOˑ is vital for understanding the role of C8-HSL MP in initiating nonspecific or innate immune responses. A concentration-dependent study was conducted to understand the optimum concentration for NOˑ release. It was determined that at a dose of 60 µg/mL, NOˑ release reached saturation when exposed to DC cells. To determine the appropriate combination of adjuvants in vaccine formulation for inducing an effective immune response, the different MPs were mixed into a heterogeneous MP suspension (Figure 8: Alum MP, MF59 MP, and CpG MP). C8-HSL MP, when combined with Alum MP + MF59 MP, produced higher NOˑ release (*p* ≤ 0.01) than when combined with CpG MP (no significance). The literature has shown that alum produces a Th2 response, MF59 produces a balanced Th1/Th2 response, and CpG produces a Th1 response [38,39]. An in vivo study is needed to determine if the C8-HSL MP adjuvant has the ability to produce a Th1 or Th2 response. Autophagy is a vital cellular catabolic process that aids in antigen presentation, secretion of cytokines, and phagocytosis [39]. We evaluated the ability of C8-HSL MP and C8-HSL MP combined with an antigen (PA MP) to induce autophagosomes. We found that when dendritic cells were exposed to C8-HSL MP combined with PA MP, there were a significantly greater number of autophagosomes than when cells were exposed to C8-HSL MP alone (Figure 6). After confirming the immunostimulatory potential of C8-HSL MP (Figure 7), we evaluated the adjuvant effect of our experimental adjuvant by combining it with two bacterial antigens and measuring NOˑ release via Griess’ assay. To determine which adjuvant combinations can be combined with the antigen, colonization factor antigen I MP (CFA/I) from *E. Coli*, for formulation of a vaccine, we combined this antigen with C8-HSL MP along with other approved adjuvants. The vaccine group, CFA MP + C8-HSL MP + Alum MP + MF59 MP, yielded the highest NOˑ release (*p* ≤ 0.001) when combined with CFA MP antigen (Figure 9). The C8-HSL MP + CFA MP group also displayed significantly higher (*p* ≤ 0.01) NOˑ release than the CFA MP group. This indicates that a vaccine formulation can be made by pairing CFA MP with C8-HSL MP + Alum MP + MF59 MP adjuvants or with C8-HSL MP only. Next, the antigen, PA MP, was combined with other adjuvants to determine which combination elicits a robust immune response (Figure 10). In the vaccine group, PA MP + C8-HSL MP yielded the highest significant response (*p* ≤ 0.001) compared with the other vaccine combinations. However, there was no significant difference (*p* > 0.05) in the PA MP + Alum MP + MF59 MP group vs. the PA MP + C8-HSL MP + Alum MP + MF59 MP group. Evaluation of the adjuvant effect was observed when different particulate vaccines were combined with our experimental adjuvant, C8-HSL MP. We tested the adjuvant effect with three specific vaccines. Zika (Figure 11) and measles vaccine MP (Figure 12) were formulated as previously described [5,19]. We also tested C8-HSL MP when combined with the marketed unadjuvanted influenza vaccine (Figure 13). Zika MP + C8-HSL MP showed a significantly higher response (*p* ≤ 0.001) than Zika MP alone, indicating that C8-HSL MP provides an additional immunological boost in response. Higher production of NOˑ by dendritic cells when exposed to Zika vaccine MPs in combination with C8-HSL MPs than when exposed to Zika vaccine MPs with any of the FDA approved adjuvants’ MPs provides more evidence of the significant adjuvant potential of our formulation. C8-HSL MP was combined with another viral antigen, measles vaccine MP, to assess adjuvant potential. The measles MP + C8-HSL MP group demonstrated a more significant response (*p* ≤ 0.001) than the antigen alone group. The measles MP + C8-HSL MP group showed higher NOˑ release when measles MP was combined with any of the other three approved adjuvants (Alum MP, MF59, and CpG MP). This study indicates that C8-HSL MP shows adjuvant potential when formulated with measles vaccine MP. The experimental adjuvant, C8-HSL MP, was combined with a marketed unadjuvanted influenza vaccine (solution). Influenza (S) + C8-HSL MP displayed significantly higher NOˑ release (*p* ≤ 0.001) than the marketed vaccine (S) when exposed to dendritic cells. Influenza vaccine (S) + C8-HSL MP showed a significantly higher response (*p* ≤ 0.001) than when the vaccine was combined with MF59 MP or CpG MP, indicating that C8-HSL can be used with the marketed influenzas vaccine. However, there was no significant difference (*p* > 0.05) between the influenza vaccine (S) + C8-HSL MP group and the influenza vaccine (S) + Alum MP group, indicating that formulating a measles vaccine can be complemented with either Alum MP or C8-HSL MP. There are limitations in this study. Investigation of several cytokines would aid in confirming the adjuvant potential of C8-HSL MP. Furthermore, an in vivo study using mice model would help to determine the cellular response. In addition, an in vivo study would help to determine if there is a significant memory response when a vaccine is combined with C8-HSL MP. Taken together, C8-HSL MP showed adjuvant potential when combined with all three viral antigens in microparticulate form. Our results indicate that the microparticulate formulation of C8-HSL has the potential to serve as an adjuvant in vaccine formulations.

## 4. Materials and Methods

### 4.1. Materials

C8-HSL, N-octanoyl-DL-homoserine lactone, was purchased from Sigma-Aldrich. Polymer poly (lactic-co-glycolic) acid (PLGA) 75:25 was purchased from Evonik Industries, Alabama, and the bovine serum albumin (BSA, Cat. A3294-50G) used to formulate microparticles was purchased from Sigma-Aldrich. Dendritic cells (DC 2.4) were purchased from ATCC. DC 2.4 cells were cultivated in Dulbecco’s Modified Eagle Medium (DMEM), purchased from ATCC. Alum and MF59^®^ were purchased from InvivoGen, San Diego, CA. CpG adjuvant was obtained through BEI Resources, NIAID, NIH: CpG 7909 Adjuvant, NR-52393. The measles vaccine was purchased from the Serum Institute of India (SII), Pune, India. Characterization of microparticles for size, charge, and polydispersity index (PDI) were measured via Zetasizer Nano ZS (Malvern Pananalytical, MA, USA). Zika virus was received by the Centers for Disease Control and Prevention (CDC), Colorado. Measles vaccines were received from the serum institute of India. Influenza vaccine was received from BEI resources. Colonization factor antigen I (CFA/I) from *E. coli.* and inactive protective antigen (PA) were received from BEI resources.

### 4.2. Methods

#### 4.2.1. Formulation of Microparticles

We formulated C8-HSL microparticles as previously described by our laboratory [19]. C8-HSL microparticles (MPs) were formulated by a W/O/W double-emulsion solvent evaporation method by using PLGA (poly (lactic-co-glycolic acid)) polymer. First, 50 mg of PLGA polymer was dissolved in 5 mL of dichloromethane (DCM). Second, C8-HSL (10 mg in 2 mL of PBS) and 150 µL of Span 80 was added to the polymer solution. Third, this solution was then homogenized (Omni THQ probe, Kennesaw, GA) for 6 cycles with 30 s on/30 s off for 2 min at 17,000 RPM to obtain the primary emulsion. Fourth, this primary emulsion was added slowly to polyvinyl alcohol (PVA, MW 30,000–70,000, Sigma-Aldrich) solution (20 mg in 20 mL of PBS) and probe homogenized (30 s on/30 s off for 2 min at 17,000 RPM) to obtain the double emulsion (W1/O1/W2). After homogenization, the emulsion was stirred for 4 hrs to allow for evaporation of the DCM. The emulsion was then ultracentrifuged (17,000 RPM, 20 min, 4 degrees) to concentrate the MPs. The resulting pellet was resuspended in trehalose (20 mg in 1 mL of PBS) as the cryoprotectant, and this concentrate was lyophilized (Labconco™ FreeZone Triad). The final microparticles were collected as lyophilized product. Adjuvant Alhydrogel^®^ MP and Zika virus MP were formulated using a similar method.

Alum, CpG adjuvant, antigens (CFA and PA), MF59^®^, and measles MP were formulated with bovine serum albumin (BSA) polymer by the spray drying method, as previously described [5]. BSA solution (1% *w/v*) was stirred and crosslinked for 24 h with glutaraldehyde (concentration of 200 µL/1 g BSA). The following day, 10 mg of sodium bisulfite was added to neutralize the glutaraldehyde. To this solution, CpG adjuvant (6% loading) was slowly added. Neutralization of glutaraldehyde by sodium bisulfite was previously determined by our laboratory [40]. A Buchi mini spray dryer B-290 was used at a rate of 15 mL/h, nozzle diameter of 0.5 mm, and nozzle temperature of −6° to yield microparticles loaded with CpG adjuvant. A similar procedure was followed for formulating microparticles for antigens (CFA and PA, 2% loading) and the measles vaccine (2% loading) [5].

#### 4.2.2. Microparticle Recovery Yield

The percentage of recovery yield of lyophilized or spray-dried product was obtained using the following formula [5,18,39]:

$$Percent\;Recovery\;yield=\frac{Weight\;of\;microparticles*100}{total\;weight\;of\;the\;formulation}$$

The microparticles were weighed using an electronic weight scale. The weight used is the percent recovery yield calculation.

#### 4.2.3. Particle Size and Zeta Potential Measurement of Microparticles

The particle size and zeta potential of the unloaded blank and the C8-HSL-loaded MPs were measured using a Malvern Nano ZS, in triplicate. This instrument is based on the principle that utilizes a laser light to illuminate the particle and is able to analyze the fluctuation in the intensity of scattered light. The principle of dynamic light scattering is that particles and molecules which are in constant random thermal motion, called Brownian motion, can diffuse at a speed related to their size. The charge acquired by a particle in a medium is its zeta potential and arises from the surface charge and is also dependent on the concentration and types of ions in the solution. Next, 2 mg microparticles were suspended in 1 mL of deionized water [1]. The size and charge of the particles were measured using a Malvern Nano ZS. The particle size of C8-HSL MP was 4.43 ± 0.29 μm. The particle sizes of all adjuvant MP ranged from 2 to 4 μm. The particle sizes of all antigens’ MPs ranged from 2 to 5 μm.

### 4.3. Morphology of Microparticles

Visualization of microparticles was conducted by utilizing scanning electron microscopy (SEM) by Phenom™ (Nanoscience instruments, Phoenix, AZ, USA) [5,18,39]. The microparticles were loaded on a single stub using double-coated carbon conductive PELCO Image Tabs™ (Ted Pella Inc., Redding, CA, USA).

### 4.4. Cytotoxicity Assay

An in vitro cytotoxicity study was conducted (as previously described) by our laboratory using the MTT (3-(4,5-dimethylthiazol-2-yl)-2,5-diphenyltetrazolium bromide) assay [5,39]. Dendritic 2.4 cells (DCs) were plated in a 96-well plate at a density of 3.5 × 10^5^ cells/well. The DC cells were exposed to increasing concentrations of C8-HSL MPs that ranged from 20 to 500 μg/mL, in triplicate, at a volume of 100 μL/well or with DMSO (50 μL/well). The 96-well plate was then incubated for 24 h at 37 °C. After 24 h, the supernatant in each well was removed, and 50 μL/well of MTT reagent (5 mg/mL in PBS) was added to all the wells in a 96-well plate. This plate, protected from light, was incubated for 4 h at 37 °C. The MTT reagent was removed before adding DMSO (dimethyl sulfoxide). After 4 h, 50 μL/well DMSO was then added to all wells to dissolve formazan. The plate was covered with foil and then placed on a shaker for 15 min at room temperature (RT). After 15 min, the plate was placed into a plate reader (BioTek Instruments, Winooski, VT, USA), and the absorbance was measured at 570 nm.

### 4.5. Number of Particles Measurement

To determine the number of particles of C8-HSL MPs in 1 mL of PBS, a laser particle counter (Spectrex PC-2200 (Redwood City, CA, USA) was used. A total of 1 mg of C8-HSL MPs was carefully weighed and placed in 1 mL of phosphate buffered saline (PBS). Measurements were repeated six times (*n* = 6).

### 4.6. In Vitro Release Study of C8-HSL

A release study of C8-HSL was conducted to determine the amount of C8-HSL that is released from the PLGA polymer. A total of 5 mg of C8-HSL MP was carefully weighed and added to an Eppendorf tube containing 1 mL of phosphate buffer saline (PBS) [18]. The Eppendorf tube was then placed in an incubator at 60 rpm at 37 °C. At specified time points (0, 1, 2, 3 to 168 h), the supernatants were collected and replaced with PBS. The sample was centrifuged at 1500 rpm for 10 min. After 10 min, the small molecule content that was released was determined via a spectrophotometer (UV) at a wavelength of 232 nm (Thermo Fisher, Waltham, MA, USA, NANODROP 2000c).

### 4.7. In Vitro Immunogenicity Griess’ Assays for Nitrite

A hallmark assessment of the induction of the innate immune response is conducted by Griess’ assay. MPs are introduced to antigen-presenting cells (APCs), which release nitric oxide (NOˑ), which can be quantified via its oxidation product, nitrite. Dendritic 2.4 cells (DCs) were plated in a 48-well plate at a seeding density of 45 × 10^4^ cells/well [5]. The next day, these DC cells were introduced to varying concentrations of C8-HSL MP. After 24 h, the supernatants were transferred to another 48-well plate, and Griess reagent (50 µL of 1% sulfanilamide in 5% phosphoric acid and 50 µL of 0.1% NED (N-1-naphthylethylenediamine dihydrochloride) in deionized water) was added. The plate was incubated in the dark for 10 min, and the absorbance was read at 540 nm using a BioTek Synergy H1 plate reader (BioTek Instruments, Winooski, VT, USA). The concentration of nitrite was calculated using a standard curve created using a 100 μM solution of sodium nitrite ranging from 0.78 to 100 μM. Next, we investigated the immunostimulatory potential of C8-HSL MP versus FDA-approved adjuvants. The DC cells were exposed to C8-HSL solution (60 μg/mL), C8-HSL MP (60 μg/mL), and C8-HSL + adjuvants (alum, MF59^®^, CpG) MP for 24 h at a dose of 15 μg/40 × 10^4^ cells). For groups that consisted of several adjuvants of MP, C8-HSL + FDA adjuvants, a dose of 20.0 μg of each adjuvant (alum, MF59, and CpG MP) was added to the DC cells. LPS served as the positive control, while the cells served as the negative control.

### 4.8. Evaluation of Adjuvant Effect: Griess’ Assay for Nitrite

To determine if C8-HSL MP can induce an adjuvant effect when combined with several antigens, Griess’ assay was conducted by assessing the release of nitric oxide from APCs. In these experiments, DCs were exposed to C8-HSL MP along with the antigens CFA, PA, measles vaccine MP, Zika virus MP, and influenza vaccine. DC 2.4 cells were plated in a 48-well plate at a seeding density of 40 × 10^4^ cells/well [5]. The next day, the cells were exposed for 24 h to the antigens CFA, PA, measles vaccine MP, zika virus MP, and influenza vaccine (solution), alone and in combination with the experimental adjuvant C8-HSL and FDA adjuvants (alum, MF59^®^, and CpG). After 24 h, the 48-well plate was placed in a plate reader (BioTek Instruments, Winooski, VT, USA) to calculate the concentration of nitrite.

### 4.9. Autophagy

An autophagy study was conducted using dendric cells (DC 2.4). DC 2.4 cells were stably transfected with the autophagy marker GFP-LC3 [39]. These DC cells were grown and plated in a 12-well plate to incubate overnight. After 24 h, Dulbecco’s Modified Eagle Medium (DMEM) was removed and the plate was washed 3 times with PBS. Then, DCs were exposed to the positive control (no exposure), negative control (rapamycin inhibitor), PA antigen (60 μg/mL), and PA antigen (30 μg/mL) + C8-HSL MP (30 μg/mL). The plate was then incubated overnight at 37 °C with 5% CO_2_. The next day, the supernatant was removed, and the cells were washed with PBS 5 times. After washing, the cells were fixed with paraformaldehyde (4% *w/v*). After fixation with paraformaldehyde, the cells were stained with DAPI (1 mg/mL) nuclear stain (Thermo Fischer Scientific, IL, USA) for 10 min. Autophagy was observed using a live cell imager Biotek (Lionheart/FX, VT, USA)**.**

### 4.10. Statistical Analysis

The statistical analysis for all experiments was performed using GraphPad Prism 5 software. The following results are expressed as the mean ± standard error of the mean (SEM). The experiments were conducted in triplicate. The comparison analysis of different experimental groups was conducted via an unpaired two-tailed t test. However, when comparing multiple groups, one-way ANOVA was utilized followed by Tukey’s post hoc test. In the graphs generated via GraphPad software, the following *p* values were used: *p* > 0.05 (ns, nonsignificant), *p* ≤ 0.05 (*), *p* ≤ 0.01 (**), *p* ≤ 0.001 (***), and *p* ≤ 0.0001 (****). In all experiments, a *p* value of less than 0.05 was considered statistically significant.

## 5. Conclusions

The microparticulate formulations of C8-HSL MP and all other formulated MPs are noncytotoxic toward dendritic cells. In vitro investigation showed that C8-HSL MP is immunostimulatory, similar to FDA-approved adjuvants such as alum, MF59, and CpG. Our experimental compound, C8-HSL MP, can be formulated with either CFA MP antigen or PA MP antigen. Additional studies with a diverse range of antigens are needed to obtain a better understanding of which adjuvant-antigen pair elicits a robust immune response. Adjuvant potential was observed when C8-HSL MP was combined with several particulate vaccines (Zika, measles, and influenza), indicating that C8-HSL MP can be combined with viral antigen as well. In vivo studies are needed to confirm the adjuvant potential and safety profile of C8-HSL MP in vaccine formulations as a potential adjuvant in both bacterial and viral vaccines.

## Figures and Tables

**Table 1:** Characterization of several adjuvants and antigens in terms of recovery yield (%), particle size (micrometers), polydispersity index (PDI), zeta potential, and number of particles/mL. Adjuvant: C8-HSL MP (N-octanoyl-L-Homoserine lactone), Alum MP (Alhydrogel^®^), MF59 (AddaVaxTM), and CpG MP 7909 (Cytosine-phosphorothioate-guanine). Antigens: colonization factor antigen (CFA) MP, protective antigen (PA) MP, Zika MP, and measles MP.

Parameter	Adjuvant			
	C8-HSL MP	Alum MP	MF59^®^ MP	CpG MP
Recovery yield	74%	85%	84%	85.6%
Particle size (μm)	4.43 ± 0.29	2.32 ± 0.42	2.58 ± 1.32	3.25 ± 0.16
Polydispersity index (PDI)	0.468 ± 0.345	0.576 ± 0.357	0.656 ± 0.735	0.345 ± 0.1674
Zeta potential (mV)	−32.0 ± 0.92	−21.1 ± 1.52	−20.2 ± 2.38	−28.0 ± 2.45
Parameter	Antigen			
	Colonization Factor Antigen (CFA) MP	Protective Antigen (PA) MP	Zika MP	Measles MP
Recovery yield	68%	70.2%	86%	84%
Particle size (μm)	3.20 ± 0.58	3.90 ± 0.38	5.71 ± 1.85	4.67 ± 0.89
Polydispersity index (PDI)	0.535 ± 0.176	0.389 ± 0.194	0.356 ± 0.158	0.485 ± 0.102
Zeta potential (mV)	−22.3 ± 1.58	22.4 ± 1.84	–25.1 ± 1.25	–19.5 ± 1.32

## Data Availability

Data are available upon request.
